# Validation and reliability for the updated REAP-S dietary screener, (Rapid Eating Assessment of Participants, Short Version, v.2)

**DOI:** 10.1186/s40795-023-00747-4

**Published:** 2023-07-19

**Authors:** Viswanathan Shankar, Kathryn H. Thompson, Judith Wylie-Rosett, C. J. Segal-Isaacson

**Affiliations:** 1 Department of Epidemiology and Population Health, Albert Einstein College of Medicine, Bronx, NY USA; 2 Department of Biomedical Sciences, College of Osteopathic Medicine, University of New England, Biddeford, ME USA

**Keywords:** Dietary screener, Nutrition, Nutrition assessment, Diet, Diet assessment, Dietary guidelines, Survey Development

## Abstract

**Background:**

The American Heart Association (AHA) chose the REAP-S dietary screener in 2020 as one of three US dietary screeners recommended for integrating dietary assessment into clinical care. The REAP-S v.2 is an updated version that is aligned with the 2020–2025 US Dietary Guidelines and is easily incorporated into electronic medical records and taught to medical students.

**Methods:**

The University of New England, Institutional Review Board, approved the study protocol. We evaluated the reliability and validity of the REAP-S v.2 scale by having first-year medical students (*n* = 167) complete both the REAP-S v.2 and a three-day food record and then analyzing their data with the following statistical techniques: Internal consistency was measured using Cronbach's alpha. Construct validity was assessed with exploratory factor analysis. Criterion validity was evaluated using analysis of variance (ANOVA) that explored the associations between REAP-S v.2 scale item responses and selected nutrient estimates from the food record analyses. The hierarchical cluster analysis classified healthy and unhealthy diet grouping under each subscale. Further using these groupings, cut points for "good" and "bad" diets for each of the three main REAP-S v.2 subscales (Food Sufficiency/Food Insufficiency; Healthy Eating Pattern and Low Nutrient Density Foods) were calculated using receiver operating characteristics (ROC) analysis. Students analyzed their three-day food intake records using an online USDA application called SuperTracker.

**Results:**

The Cronbach’s alpha measuring internal consistency was acceptable for the overall scale at 0.71. The exploratory factor analysis extracted three factors that roughly paralleled the three main subscales, suggesting construct validity. Most selected food record-derived nutrient values were significantly associated with scale items confirming criterion validity. The score cut points suggest that dietary counseling might be needed at ≤ 8, ≤ 10, and ≤ 16 for the above subscales.

**Conclusion:**

The REAP-S v.2 is intended for clinicians to use as a brief dietary screener with their patients. Tested in a population of first-year medical students, the REAP-S v.2 brief dietary screener showed acceptable internal consistency, criterion, and construct validity. It is easily scored and incorporated into the electronic medical record.

**Supplementary Information:**

The online version contains supplementary material available at 10.1186/s40795-023-00747-4.

## Background

In 2020 the American Heart Association (AHA) recommended that clinicians use one of three dietary screeners to evaluate patients’ dietary habits regarding cardiovascular disease risk reduction [1]. The original REAP-S (Rapid Eating Assessment for Participants, short version, [2]) was one of the AHA’s three recommended dietary screening tools. In this paper, we present validity and reliability analyses for an updated and revised version of the REAP-S dietary screener.

Eleven million deaths worldwide in 2017 were linked to people eating poor diets high in sugar, salt, and processed meat, which contributed to heart disease, cancer, and diabetes [3]. The AHA stated in their 2020 position paper on dietary screeners that “It is critical that diet quality be assessed and discussed at the point of care with clinicians and other members of the healthcare team to reduce the incidence and improve the management of diet-related chronic disease, especially cardiovascular disease” [1]. Suffering and expense caused by nutrition-related chronic diseases have significantly increased during this half-century, but physician competency in nutrition has not [4]. There are many complex reasons for this discrepancy. Funding models rewarded disease-oriented interventions and treatments rather than lifestyle interventions to maintain health and prevent disease, contributing to the “disturbing mismatch between the skills of physicians and the needs of patients” concerning nutrition [5].

A 2018 AHA science advisory, which focused on nutrition training for physicians, has optimistically stated: “Enhancing physician education and training in nutrition, as well as increasing collaborative nutrition care delivery by 21st-century health systems, will reduce the health and economic burdens from atherosclerotic cardiovascular disease to a degree not previously realized” [6].

A two-pronged approach is needed to equip physicians and other healthcare providers with the training and screening tool to assure that “diet quality (is) assessed and discussed at the point of care with clinicians and other members of the healthcare team to reduce the incidence and improve the management of diet-related chronic disease, especially cardiovascular disease” [7]. By gaining this experience early, physicians may be more likely to use a screener to assess patients’ dietary behaviors and refer them to registered dietitian nutritionists as needed. 2) Both medical systems and private practitioners should be encouraged to incorporate a simple dietary screener into an initial patient evaluation screening. The data are then entered into the patient’s electronic medical record.

The REAP-S is a brief dietary screening tool designed to provide clinicians who may want to take a quick snapshot of the strengths and weaknesses of a patient’s diet. It is intended to be administered before the patient sees the primary care provider (PCP) so the PCP can use the information to counsel or refer the patient as necessary. The screener can be easily entered into an electronic medical record system and scored automatically.

The original REAP-S dietary screener was first published in 2004 [2]. We recently updated it to 1) Reflect current recommendations in the Dietary Guidelines for Americans 2020–2025 [8], 2) Make it easier for patients to identify portion sizes [9], and 3) Provide clinicians empirically tested cut points for REAP-S v.2 scores. For clarity, we refer to the original REAP-S as REAP-S v.1 and the updated version as REAP-S v.2. This paper describes the validity and reliability study for REAP-S v.2.

## Methods

### Development of the REAP-S v.2 dietary screener

As previously mentioned, REAP-S v.2 was developed to give clinicians a rapid assessment of a patient’s dietary adequacy, including an evaluation of healthy eating and exercise patterns and the consumption of foods that should be limited (e.g., added sugars, saturated fats, sodium, and alcohol).

To do this, the questions in REAP-S v.1 were reorganized into these four subscales: Food Sufficiency/Food Insufficiency; Healthy Dietary Pattern; Low Nutrient Density Foods; and Exercise, and several questions were added or modified. The REAP-S v.2 instrument is presented in the supplemental material.

Questions in the Food Sufficiency/Food Insufficiency subscale (shown in blue) provide information on total caloric intake, protein intake, and calcium intake. Questions 1 and 4 are from REAP–S v.1. Questions 2 and 3 were added for protein and calorie intake information. In the Healthy Eating Pattern subscale (pink), Questions 5–7 were modified from REAP-S v.1 to include more descriptive examples of portion sizes. Questions 8–11 were added to the Healthy Eating Pattern subscale to include the intake of healthy fats found in vegetable oils, nuts and fish and the complex carbohydrates and fiber found in legumes, reflecting more recent dietary recommendations. In the Low Nutrient Density Foods subscale (green), the order of the questions was changed, and some of the questions were modified to include descriptive examples of portion sizes. Question 19 was added to provide information on alcohol intake. Question 20 in the Exercise subscale (yellow) was added to provide information on physical activity. We included question 21 from REAP-S v.1 to provide a segue for beginning the conversation on readiness to change. The subscale scores provide the clinician with the option to acknowledge areas of strength and focus on the most problematic areas for patients who are ready to make changes.

### Study population

All students who participated in this study were in the first-year Osteopathic Medical Knowledge Course at the University of New England. Completion of the REAP-s v.2 and the three-day food record were part of a larger required project called the Health Promotion Project. The participant demographic data collected by the College of Osteopathic Medicine as part of the admissions process was used to describe the study population. The deidentified data included students’ gender, age, and self-reported race/ethnicity**.**

### Study protocol

During the first week of the Health Promotion Project assignment, students were invited to participate in a study to validate a new dietary screener, REAP-S v.2. The course resource materials included a written description of the validation study. The data collected for the validation study were part of the University of New England College of Osteopathic Medicine curriculum activities for all students. Students who did not wish to participate in the validation study were given an opt-out form to complete and their data were not included. The University of New England Institutional Review Board approved the study protocol.

**Fig. 1:**
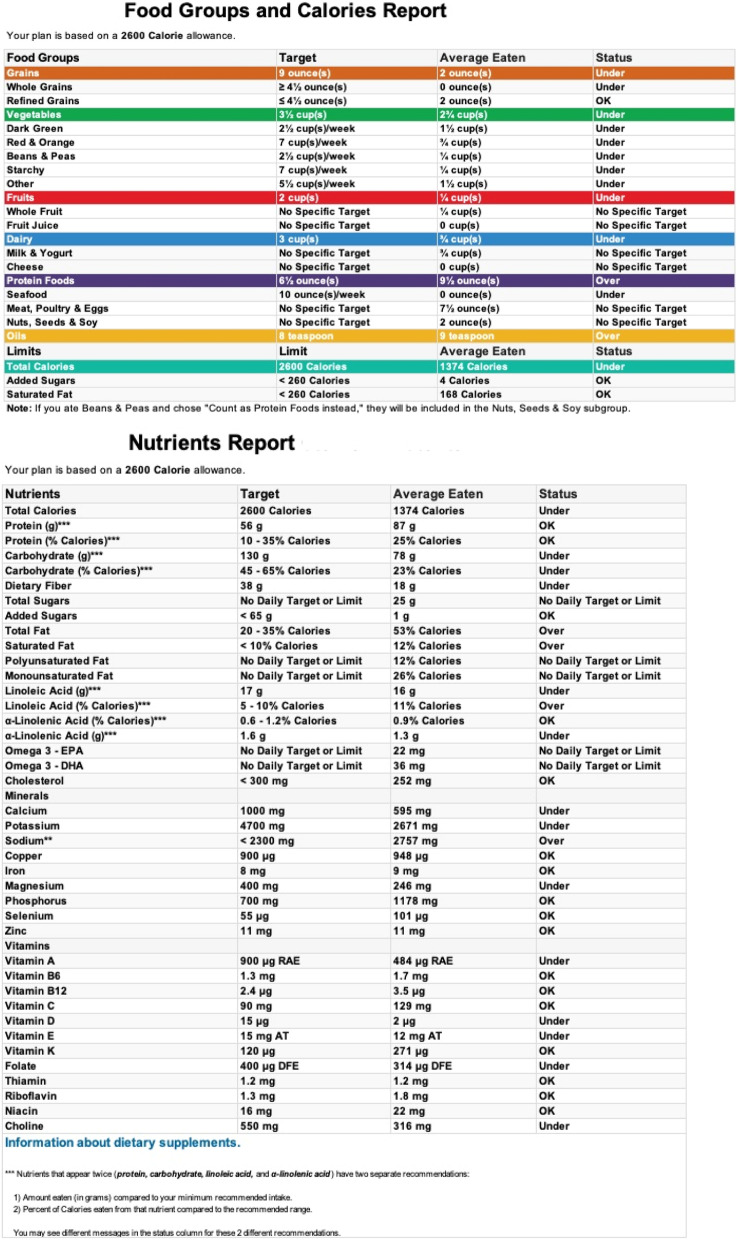
Example of output from SuperTracker for a three day diet record

As part of the assignment, students were required to complete an assessment of their diet by keeping a three-day diet record consisting of two weekdays and one weekend day. Students entered their food records into an online United States Department of Agriculture (USDA) diet planning and tracking tool, SuperTracker [10], to calculate their individual nutrient and food group intake which were averaged over the three days of reported intake (Fig. 1). Students also completed the revised REAP-S v.2 dietary screener using an online form. SuperTracker analysis data and the REAP screener data were deidentified and entered into a Microsoft Excel spreadsheet [11]. No students chose to opt out of the study, although some did not complete all parts of the assignment. It wasn’t possible to do test–retest reliability because the students’ curriculum did not allow for it.

### Statistical analysis

Participant characteristics, dietary data analyzed with SuperTracker, and responses to REAP-S v.2 were numerically summarized using descriptive statistics. The continuous variables were summarized using mean and standard deviation, while categorical variables were presented as frequency counts and percentages. The scale’s internal consistency was measured using Cronbach’s alpha, which assesses how individual items correlate with other items and the total scale.

The REAP-S v.2’s validity was assessed as follows. Exploratory factor analysis (EFA) was used to identify the construct validity (factors), nature, and number of constructs. The Kaiser–Meyer–Olkin (KMO) test was used to examine the sampling adequacy of the scale. A common factor analysis with an iterated principal factor extraction method and a Promax rotation was used, assuming the factors are correlated. Three factors were extracted based on the majority rule. The process included six methods: Eigenvalue > 1 criterion, Kaiser minimum Eigenvalue criteria, the cumulative proportion of variance (0.8), parallel analysis [12], minimum average partial test (MAP) [13], and the visual scree test [14]. The replicability of the estimates was examined using the Bootstrap estimates and 95% confidence interval procedure. The bootstrap analyses were performed based on 2000 replicated data.

Furthermore, an analysis of variance (ANOVA) was fitted to explore the associations between REAP-S v.2 scale items and selected macronutrients, minerals, and vitamins measured from the averaged three-day food records analyzed with SuperTracker. Some non-normally distributed variables were log-transformed to conform to a Gaussian distribution. A score from the analysis of participants’ dietary records for healthy versus unhealthy eating habits was not readily available; we used a nutrient marker provided by the participants’ SuperTracker analyses to derive an objective measure for healthy eating habits. A hierarchical cluster analysis was used on the selected nutrients for each REAP-S v.2 subscale to create two clusters representing healthy versus unhealthy eating behaviors. Each REAP-S subscale’s best optimal cut point scores were then ascertained by modeling the derived health status (healthy/unhealthy) as an outcome variable, using receiver operating characteristics (ROC) curves via a logistic regression model.

*Missing Data:* Participants (*n* = 9) who did not complete the REAP-S v.2 and Super Tracker were excluded from the analysis. Others with random incomplete information were imputed using a fully conditional imputation approach. All analyses were performed using SAS software version 9.4 [15].

## Results

### Population sample

In 2017, 178 students enrolled in the University of New England College of Osteopathic Medicine, of whom 176 participated in the study. Fifty-one percent (*n* = 90) were male; the average age was 24 years (min, max: 20, 39). About 68% were New England state residents, 29.7% were from other US states, and 2.2% were international. Racially, 71% self-reported as White, 25.8% as of Asian or Middle Eastern race, 2.8% were the underrepresented minority, and 5.6% were unknown. Of the 176 participants, nine did not complete the instrument, thus reducing the analysis sample to 167.

### Reliability and validity of the scale

Descriptive statistics of the scale items are presented in Table 1. The average total (SD) REAP score was 39.08 (6.37), approximately the mid-point of the overall total score. The mean (SD) of Food Sufficiency/Food Insufficiency, Healthy Eating Patterns, Low Nutrient Density Foods, and Exercise subscale score in our study population were 9.42(1.83), 10.98(3.27), 16.81 (3.22) and 2.17(0.85), respectively. Nearly 28% (*n* = 46) of the sample reported a score suggestive of food insufficiency (Food Sufficiency / Insufficiency scale ≤ 8 scores); 87% (*n* = 143) reported unhealthy eating patterns (Healthy Eating Patterns ≤ 14 scores), 46% (*n* = 76) reported not consuming low nutrient density foods (Low Nutrient Density Foods ≤ 16 scores) and 25% (*n* = 41) reported less than 15 min a day of exercise on fewer than three days a week (Exercise ≤ 1 score). Ninety percent of the participants reported wanting to change eating and physical activity habits.

**Table 1:** Descriptive statistics: *n* = 167. Mean, Standard Deviations, Factor Loadings and Bootstrap estimates with 95% CI and Communality (h^2^) from Iterated principal factor analysis with Promax Rotation

Item statement: In an average week, how often do you:	Item code	Mean (SD)	Rotated Structure (correlations)			Rotated Factor Pattern (Standardized Regression Coefficients) (Loadings)			Bootstrap Factor Pattern Estimates (95% CI)			h^2^ 95% CI
			**F1**	**F2**	**F3**	**F1**	**F2**	**F3**	**F1**	**F2**	**F3**	
1. Not feel well enough to shop or cook?	Q_1	2.30 (0.78)	0.35	0.31	0.20	0.24	0.23	0.14	0.2 (-0.01, 0.55)	0.2 (-0.12, 0.47)	0.13 (-0.18, 0.55)	0.24 (0.08, 0.41)
2. Eat fewer than two meals per day?	Q_2	2.71 (0.58)	0.22	0.07	**0.35**	0.14	0.01	**0.32**	0.16 (-0.11, 0.47)	-0.02 (-0.33, 0.21)	0.29 (-0.07, 0.68)	0.18 (0.05, 0.41)
3. Eat less than 3 oz per day (see sizes below) of high protein foods such as poultry, meat, fish, tofu, 1 oz. nuts or 1½ cups of beans?	Q_3	2.35 (0.79)	0.11	-0.15	**0.68**	0.00	-0.18	**0.68**	0.12 (-0.17, 0.45)	-0.19 (-0.44, 0.12)	0.48 (-0.05, 0.78)	0.35 (0.09, 0.68
4. Consume less than 2 servings of a calcium-rich food such as milk, yogurt, cheese, calcium-fortified soy, rice or almond milk?	Q_4	2.06 (0.86)	0.02	0.03	**0.45**	-0.12	0.05	**0.48**	-0.01 (-0.35, 0.31)	-0.00 (-0.31, 0.27)	0.35 (-0.13, 0.79)	0.22 (0.02, 0.59)
5. Eat 3 or more servings of vegetables per day?	Q_5	1.71 (0.81)	**0.66**	0.42	0.24	**0.56**	0.23	0.09	**0.55** **(0.26, 0.73)**	0.20 (-0.10, 0.44)	0.09 (-0.32, 0.52)	0.50 (0.35, 0.63)
6 Eat 2 or more servings of fruit per day? *(Do not include fruit juice or fruit drinks.)*	Q_6	1.67 (0.87)	**0.50**	0.18	0.16	**0.48**	0.02	0.04	**0.45** **(0.16, 0.66))**	0.04 (-0.16, 0.27	0.08 (-0.31, 0.46)	0.29 (0.12, 0.50)
7. Eat 2 or more servings of whole grain products or high fiber starches a day?	Q_7	2.21 (0.84)	**0.44**	-0.11	-0.01	**0.57**	-0.29	-0.14	**0.45** **(0.07, 0.71)**	-0.20 (-0.42, 0.02)	-0.04 (-0.36, 0.39)	0.27 (0.06, 0.53)
8. Eat fish, shellfish or other seafood?	Q_8	0.84 (0.62)	**0.29**	-0.07	0.11	**0.35**	-0.19	0.03	0.29 (-0.1, 0.52)	-0.13 (-0.50, 0.14)	0.07 (-0.34, 0.48)	0.17 (0.04, 0.37)
9. Eat beans, peas, lentils or other legumes?	Q_9	1.00 (0.74)	**0.38**	0.28	-0.01	**0.35**	0.16	-0.10	**0.32** **(0.07, 0.54)**	0.15 (-0.09, 0.41)	-0.06 (-0.35,0.27)	0.20 (0.07, 0.37)
10. Eat tree nuts, peanuts or nut butters?	Q_10	1.54 (0.92)	**0.56**	0.26	0.03	**0.56**	0.08	-0.11	**0.49** **(0.11, 0.75)**	0.11 (-0.18, 0.35)	-0.02 (-0.37, 0.38)	0.35 (0.17, 0.58)
11. Use olive oil, peanut oil or other vegetable oils?	Q_11	1.97 (0.86)	**0.42**	0.16	0.07	**0.42**	0.02	-0.03	**0.40** **(0.13, 0.62)**	0.03 (-0.18, 0.23)	-0.00 (-0.30, 0.37)	0.21 (0.07, 0.39)
12. Eat high fat meats such as hamburger, ribs, steak, lamb chops, chicken or turkey wings, hot dogs or cold cuts such as bologna and salami	Q_12	1.86 (0.86)	0.16	**0.51**	-0.18	0.04	**0.50**	-0.21	0.04 (-0.19, 0.44)	**0.44** **(0.06, 0.72)**	-0.22 (-0.8, 0.15)	0.38 (0.14, 0.70))
13. Eat more than 1 tablespoon of cooking or table fats that are solid at room temperature such as butter, stick margarine, bacon fat or vegetable shortening (like Crisco™)?	Q_13	2.10 (0.85)	0.10	**0.52**	-0.12	-0.04	**0.53**	-0.13	-0.01 (-0.24, 0.26)	**0.48** **(0.06, 0.72)**	-0.17 (-0.67, 0.15)	0.35 (0.14, 0.58)
14. Drink 12 oz or more of non-diet soda, fruit drink/punch, fruit juice or Kool-Aid™ per day? 1 can of soda = 12 oz	Q_14	2.66 (0.71)	0.18	**0.52**	0.03	0.01	**0.52**	0.01	0.07 (-0.18, 0.38)	**0.44** **(0.06, 0.67)**	-0.01 (-0.48, 0.51)	0.30 (0.14, 0.52)
15. Eat sweets like cake, cookies, pastries, donuts, toaster pastries, muffins, chocolate and candies	Q_15	1.75 (0.83)	0.17	**0.56**	0.03	-0.02	**0.56**	0.02	0.04 (-0.14, 0.57)	**0.51** **(0.05, 0.73)**	-0.01 (-0.48, 0.51)	0.35 (0.19, 0.59)
16. Eat packaged snack foods such as chips, salted pretzels, pizza bites, etc	Q_16	1.96 (0.80)	0.11	**0.56**	0.07	-0.10	**0.59**	0.07	-0.03 (-0.32, 0.51))	0.53 (-0.04, 0.82)	0.06 (-0.35, 0.74)	0.39 (0.15, 0.70)
17. Eat meals from restaurants, take-out places, convenience stores or entertainment venues?	Q_17	2.03 (0.66)	0.29	**0.46**	0.29	0.08	**0.42**	0.25	0.15 (-0.14, 0.49)	**0.34** **(0.01, 0.60)**	0.20 (-0.47, 0.59)	0.33 (0.15, 0.53)
18. Prepare meals at home from basic ingredients such as fresh or frozen vegetables, uncooked poultry, pasta, beans etc.?	Q_18	2.15 (0.80)	**0.52**	0.16	0.39	**0.45**	0.00	0.28	**0.45** **(0.08, 0.70)**	-0.00 (-0.23, 0.21)	0.25 (-0.11, 0.57)	0.37 (0.21, 0.53)
19. Have more than 1 alcoholic drink per day if you're a woman or 2 alcoholic drinks per day if you're a man?	Q_19	2.31 (0.57)	-0.08	0.07	-0.05	-0.11	0.10	-0.03	-0.09 (-0.33, 0.19)	0.09 (-0.13, 0.34)	0.0 (-0.34, 0.42)	0.06 (0.0, 0.19)
20. Walk for at least one mile (about 2000 steps) or exercise for at least 15 min?	Q_20	2.17 (0.85)	**0.37**	0.17	0.24	**0.30**	0.07	0.16	0.32 (-0.04, 0.53)	0.06 (-0.23, 0.38)	0.14 (-0.35, 0.47)	0.21 (0.09, 0.39)

F1: Healthy Dietary Pattern, F2: Low Nutrient Density Foods, F3: Food Inadequacy/Food Insufficiency

The estimate of the internal consistency of the overall scale as measured by Cronbach’s alpha is 0.71, which is within acceptable limits for all study variables (i.e., α ≥ 0.70). Internal consistency estimates for the responses to the subscales; Food Sufficiency/Food Insufficiency, Healthy Eating Pattern and Low Nutrient Density Foods subscales were respectively α = 0.41, α = 0.65, and α = 0.62.

An exploratory factor analysis was conducted from the data collected from the 167 participants using the iterated principal factor analysis with Promax rotation. The KMO index measuring the sampling adequacy for each variable in the model was 0.67, indicating the sample was adequate to perform factor analysis. Three factors were extracted based on the majority criteria of extraction methods described in the statistics section. The rotated factor pattern (standardized regression coefficients or loadings) of items, factor structure (correlations), Bootstrap estimates of rotated factor pattern with 95% confidence intervals, and communality estimates are presented in Table 1. The rotated pattern matrix and rotated structure matrix support the three-factor solutions. The structure matrix values show the correlation between items and factors.

In contrast, the pattern matrix value presents a relationship between items and factors while holding other factors constant, hence the value differences. These three factors explained 87.49% of the total variance. The three factors loaded similarly to the theoretical constructs with minor changes. Factor 1, the Healthy Eating Pattern vector, consisted of nine items with factor loadings ranging from 0.31 to 0.59. Factor 2, the Low Nutrient Density Foods vector, had factor loadings from 0.43 to 0.56, and Factor 3, the Food Sufficiency/Food Insufficiency vector, had loadings from 0.33 to 0.66. Alcohol and feeling well did not play a major role in these constructs, possibly due to the age group of the study participants. The replicability of these factors was assessed through Bootstrap estimates with 2000 replications, which mainly showed similar trends. After ProMax rotation, the factors remain correlated. Table 2(a) presents inter-factor correlations between factor 1 vs. factor 2, factor 1 vs. factor 3, and factor 2 vs. factor 3 were 0.34, 0.25 and 0.04, respectively. Each item's proportion of variability explained by the factors assessed in terms of communality statistics ranged between 0.17 to 0.5, except for alcohol. The communality contribution for alcohol was very low, 0.06, consistent with its lack of loading on the factors. Table 2 (b-c) presents summary statistics for two types of factor scores: rotated factor pattern loadings and standardized loadings. From the results, though the two scores are different in metrics, the correlation table shows the factors provide similar results: factor 1 is correlated with two other factors, while factor 2 is uncorrelated with factor 3. Each subscale showed adequate psychometric properties independently and could be used separately.

**Table 2:** Inter-Factor correlation, descriptive statistics, and correlation between factor scores

	**Factor 1 (Healthy Dietary Pattern)**		**Factor 2 (Low Nutrient Density Foods)**		**Factor 3 (Food Sufficiency/ Insufficiency)**	
**(a) Inter-Factor Correlations**						
Factor 1 (Healthy Dietary Pattern)	1.00		0.34		0.25	
Factor 2 (Low Nutrient Density Foods)	0.34		1.00		0.04	
Factor 3 (Food Sufficiency/ Insufficiency)	0.25		0.04		1.00	
**(b) Descriptive statistics of factor scores**						
	**Mean (SD)**		**Min, Max**		**Sum**	
Factor 1 (Healthy Dietary Pattern)	0 (0.87)		-2.35, 2.21		0	
Factor 2 (Low Nutrient Density Foods)	0 (0.86)		-3.37, 1.72		0	
Factor 3 (Food Sufficiency/ Insufficiency)	0 (0.80)		-2.45, 1.49		0	
Pattern Factor 1	0.37 (0.10)		0.08, 0.63		61.50	
Pattern Factor 2	0.35 (0.09)		-0.01, 0.53		58.21	
Pattern Factor 3	0.22 (0.06)		0.05, 0.32		36.15	
**(c) Correlations between set of factor scores**						
	**Factor 1**	**Factor 2**	**Factor 3**	**Pattern Factor 1**	**Pattern Factor 2**	**Pattern Factor 3**
Factor 1 (Healthy Dietary Pattern)	1.00	0.42	0.33	0.99	0.34	0.27
Factor 2 (Low Nutrient Density Foods)	0.42	1.00	0.04	0.35	0.99	0.001
Factor 3 (Food Sufficiency/ Insufficiency)	0.34	0.04	1.00	0.30	0.008	0.99
Pattern Factor 1*	0.99	0.35	0.30	1.00	0.27	0.23
Pattern Factor 2*	0.34	0.99	0.008	0.27	1.00	-0.03
Pattern Factor 3*	0.27	0.008	0.99	0.23	-0.03	1.00

Patten Factor # – are rotated pattern coefficients; Factor # are standardized scoring coefficients

### Associations between the REAP-S v2. scale items and food record dietary data

We tested how well each scale item was associated with and predicted specific macronutrients, minerals, vitamins, and food groups estimated from the participants' three-day dietary self-report and compiled and analyzed with Super Tracker. Some non-normally distributed variables were log-transformed to conform to a Gaussian distribution. The association results for each scale item are presented in Table 3. Most macronutrients, minerals, vitamins, and food groups were statistically associated with scale items. These selected nutrients and food groups that were associated with each of the subscales were used to differentiate participants into cluster groups of healthy and unhealthy eating patterns using hierarchical cluster analysis. The dendrogram, a branching diagram, shows the relationship of similarity among participants based on the participant-reported SuperTracker nutrient variables values forming two clusters (healthy and unhealthy behavior) generated based on the cluster analysis (Supplemental Fig. 1a-c). Further, these groupings were tested against the REAP-S v.2’s subscale scores. The ROC analysis showed subscale cut points of ≤ 8, ≤ 10, ≤ 16, and ≤ 1, respectively, for the Food Sufficiency/Food Insufficiency subscale; Healthy Eating Pattern subscale; Low Nutrient Density Foods subscale; and Exercise to distinguish those who might benefit from nutrition counseling from those who should be congratulated on their healthy behaviors.

**Table 3:** Relationship between REAP-S v.2 scale items and selected nutrients

**Scale Item**	**Outcomes (Nutrients/Minerals/Vitamins)**	**Every Day**	**More Than 2 Times A Week**	**2 Or Fewer Times A Week**	**Never**	***p*** **Value**
**Food Insufficiency/Food Sufficiency**						
Not feeling well	Log (total calories) g	5.40 (0.42)	5.25 (0.24)	5.33 (0.26)	5.30 (0.30)	0.6579
	Log(protein) g	4.68 (0.60)	4.33 (0.60)	4.47 (0.31)	4.48 (0.43)	0.2447
Eat < 2 meals per day	Log (total calories) g	-	5.3 (0.24)	5.28 (0.25)	5.31 (0.29)	0.8432
	Log(protein) g	-	4.44 (0.40)	4.44 (0.28)	4.47 (0.40)	0.9032
Eat less than 3 oz per day of high protein	Log(protein) g	4.31 (0.46)	4.38 (0.39)	4.32 (0.30)	4.58 (0.39)	0.0004
	Log (iron) mg	3.10 (0.65)	2.75 (0.41)	2.66 (0.35)	2.76 (0.39)	0.0766
Consume less than 2 servings of a calcium-rich food	Calcium mg	846.88 (361.17)	833.38 (373.54)	922.88 (312.56)	1053.21 (371.34)	0.0274
	Log(vitamin D) mcg	1.36 (0.79)	1.58(0.73)	1.59 (0.65)	1.64 (0.62)	0.7230
**Healthy Dietary Pattern**						
Eat 3 or more servings of vegetables per day	Vegetable cups	3.20 (1.21)	2.52 (0.98)	1.92 (1.06)	1.15 (0.65)	< 0.0001
	Log(Vitamin C)	4.84 (0.68)	4.61 (0.54)	4.17 (0.70)	4.08 (0.16)	< 0.0001
	Log (folate)	6.38 (0.53)	6.37 (0.38)	6.33 (0.44)	6.12 (0.40)	0.6897
	Log (potassium)	8.08 (0.34)	7.97 (0.29)	7.80 (0.36)	7.88 (0.30)	0.0005
	Log (dietary fiber)	3.25 (0.49)	3.17 (0.32)	2.95 (0.43)	2.88 (0.23)	0.0010
Eat 2 or more servings of fruit per day?	Fruit cups	2.09 (1.16)	1.46 (0.79)	0.95 (0.86)	0.33 (0.34)	< 0.0001
	Log(Vitamin C)	4.71 (0.81)	4.60 (0.55)	4.27 (0.69)	4.01 (0.55)	0.0006
	Log (folate)	6.38 (0.46)	6.44 (0.40)	6.27 (0.44)	6.13 (0.45)	0.0414
	Log (potassium)	8.06 (0.34)	7.96 (0.32)	7.85 (0.33)	7.65 (0.35)	0.0008
	Log (dietary fiber)	3.22 (0.45)	3.17 (0.41)	2.99 (0.40)	2.78 (0.26)	0.0012
Eat 2 or more servings of whole grain products or high fiber starches a day?	Log (whole grain)	1.06 (0.51)	0.83 (0.45)	0.56 (0.51)	0.54 (0.76)	< 0.0001
	Log (folate)	6.42 (0.46	6.30 (0.46)	6.30 (0.27)	6.04 (0.48)	0.1414
	Log (dietary fiber)	3.15 (0.42)	3.10 (0.41)	2.94 (0.46)	2.86 (0.23)	0.0913
Eat fish, shellfish or other seafood?	Log (seafood)	2.52 (2.02)	1.44 (1.19)	1.01 (1.04)	0.28 (0.60)	0.0635
	Log (iron) mg	3.15 (0.36)	2.84 (0.60)	2.70 (0.35)	2.78 (0.40)	0.1929
Eat beans, peas, lentils or other legumes?	Log(beans)	0.80 (0.67)	0.41 (0.46)	0.21 (0.33)	0.11 (0.17)	< 0.0001
	Log(iron) mg	3.17 (0.49)	2.70 (0.36)	2.72 (0.39)	2.74 (0.39)	0.0562
	Log(dietary fiber)	3.77 (0.61)	3.21 (0.44)	3.07 (0.39)	2.92 (0.33)	< 0.0001
Eat tree nuts, peanuts or nut butters?	Log(nuts)	0.80 (0.83)	0.25(0.80)	0.13 (0.81))	-0.16 (0.70)	0.0019
	Monounsaturated Calories gm	1.35 (0.30)	1.42 (0.40)	1.48 (0.31)	1.21 (0.39)	0.0328
	Saturated Fat calories gm	1.16 (0.44)	1.20 (0.35)	1.28 (0.39)	1.08 (0.34)	0.1780
	Log (total calories) gm	5.40 (0.35)	5.32 (0.25)	5.28 (0.26)	5.22 (0.29)	0.1267
Use olive oil, peanut oil or other vegetable oils?	Oil Tsp	5.25 (2.61)	5.16 (2.60)	4.76 (2.76)	4.50 (2.59)	0.7474
	Monounsaturated Calories gm	1.47 (0.33)	1.47 (0.37)	1.28 (0.32)	1.09 (0.41)	0.0021
	Saturated Fat calories gm	1.28 (0.45)	1.23 (0.38)	1.15 (0.26)	0.94 (0.36)	0.1141
**Low Nutrient Density**						
Eat high fat meats	Log (meat, poultry, egg)	1.71 (0.73)	1.52 (0.64)	1.27 (0.62)	0.82 (0.81)	0.0381
	Saturated Fat calories gm	1.21 (0.36)	1.33 (0.29)	1.21 (0.42)	1.08 (0.37)	0.0284
	Total Fat Calories gm	3.82 (0.83)	3.84 (0.60)	3.90 (0.90)	3.57 (0.64)	0.1948
	Square Root (Cholesterol)	21.51 (8.72)	18.63 (5.14)	16.94 (4.84)	14.72 (5.08)	0.0007
	Log (protein) gm	4.64 (0.52)	4.56 (0.33)	4.48 (0.37)	4.29 (0.34)	0.0025
Eat more than 1 tablespoon of cooking or table fats	Saturated Fat calories gm	1.13 (0.43)	1.38 (0.47)	1.25 (0.32)	1.10 (0.36)	0.0060
	Square Root (Cholesterol)	19.48 (7.44)	17.03 (4.29)	16.85 (4.88)	17.20 (6.93)	0.6840
Drink 12 oz or more of non-diet soda, fruit drink/punch, fruit juice or Kool-Aid per day?	Total sugars gm	98.60 (33.78)	99.88 (38.26)	82.88 (33.97)	70.60 (30.65)	0.0103
	Carbohydrate gm	270.40 (47.45)	253.50 (57.50)	225.60 (56.62)	212.76 (71.04)	0.1072
	Log (total calories) gm	5.43 (0.13)	5.48 (0.15)	5.34 (0.23)	5.29 (0.30)	0.1590
Eat sweets	Total Sugar gm	73.30 (28.50)	80.62 (37.19)	74.69 (29.9)	66.03 (30.57)	0.2839
	Added sugar	144.82 (50.58)	138.80 (90.94)	119.84 (76.15)	74.83 (50.26)	0.0026
	Log (total calories) gm	5.30 (0.16)	5.33 (0.27)	5.28 (0.28)	5.35 (0.34)	0.5854
	Saturated Fat calories gm	1.34 (0.40)	1.28 (0.34)	1.16 (0.37)	1.16 (0.45)	0.2022
	Total Fat Calories gm	3.94 (0.79)	3.88 (0.74)	3.73 (0.70)	3.76 (0.97)	0.6827
Eat packaged snack foods	Log (sodium)	8.19 (0.22)	7.98 (0.34)	7.90(0.31)	7.87(0.50)	0.2379
	Log (total calories) gm	5.40 (0.19)	5.33 (0.24)	5.30 (0.25)	5.30 (0.37)	0.8664
Eat meals from restaurants	Log (total calories) gm	5.68 (0.20)	5.26 (0.27)	5.32 (0.25)	5.28 (0.36)	0.2116
	Log (sodium)	8.67 (0.23)	7.96 (0.37)	7.93 (0.33)	7.81 (0.45)	0.0083
	Total Fat Calories gm	3.39 (1.02)	3.67 (0.68)	3.87 (0.81)	3.72 (0.73)	0.4736
	Saturated Fat calories gm	1.06 (0.55)	1.19 (0.34)	1.22 (0.38)	1.21 (0.44)	0.9189
	Total sugars gm	81.50 (54.45)	68.0 (40.04)	74.77 (28.06)	78.49 (35.77)	0.6254
Prepare meals at home	Log (total calories) gm	5.03 (0.25)	5.29 (0.24)	5.29 (0.26)	5.37 (0.30)	0.0353
	Log (sodium)	7.54 (0.49)	7.87 (0.38)	7.96 (0.35)	7.94 (0.38)	0.0533
	Saturated Fat calories gm	3.09 (0.81)	3.68 (0.83)	3.84 (0.72)	3.86 (0.78)	0.1021
	Log (dietary fiber) gm	2.68 (0.35)	3.08 (0.36)	3.02(0.40)	3.21(0.44)	0.0053
Have more than 1 alcoholic drink per day	Log (total calories) gm	-	5.39 (0.20)	5.31(0.27)	5.29(0.30)	0.6035
	Log (alcohol)	-	3.0 (2.26)	1377 (2.17)	0.39(1.15)	0.1083
Walk for at least one mile	Log (total calories) gm	5.37(0.28)	5.27(0.29)	5.22(0.15)	5.42 (0.15)	0.0334

## Discussion

### The use of dietary screeners

Dietary behaviors are one of the most difficult human behaviors to measure accurately. Many individuals may not accurately recall what or how much they ate or drank. Social desirability may influence some to report an intake they perceive as healthier. Just the act of self-observation, as in the case of self-completed food records, may affect intake. Food intake from day to day and week to week may vary for many reasons, such as access to food, health, travel, changes in routine, etc. Several methods have been developed and tested that show good reliability and validity for measuring food intake for research studies. Some examples of these are semi-quantitative food frequency questionnaires and multiple-pass, multiple-day food recalls administered by a trained research dietitian. Krebs-Smith et al. has developed an excellent resource on dietary intake research tools. The resource is available online at the National Cancer Institute’s website at https://dietassessmentprimer.cancer.gov/ [16].

As mentioned earlier, dietary screeners, such as REAP-S v.2, are intended to give clinicians a quick and rough snapshot of a patient’s dietary patterns. As useful as dietary screeners are, they are not a substitute for nutrition research instruments.

### Summary of results

The overall REAP-S v.2 screener showed an acceptable internal consistency comparable to the original REAP-S v.1 (Cronbach’s alpha of 0.72) using a similar sample of first-year medical students [17]. The total REAP score ranged between 25 to 55 (max = 80), while each subscale score range was food sufficiency/insufficiency score (max = 12): 3 to 12, healthy eating patterns (max = 21): 1 to 20, low nutrient density foods (max = 24): 6 to 24, and exercise (max = 3): 0 to 3. The standardized factor scores have a mean zero and variability closer to one.

The constructs identified in exploratory factor analysis paralleled the proposed three main subscales of the REAP-S v. 2: Factor 1, the Healthy Eating Patterns vector; Factor 2, the Low Nutrient Density Foods vector and Factor 3, the Food Sufficiency/ Food Insufficiency vector. The questions on feeling well and alcohol intake did not load well in these factors, which could be due to the specific sample group.

Criterion validity was evaluated by comparing selected nutrition data from averaged three-day food records to REAP-S v.2 scale items. Most nutrient data were significantly associated with scale items, confirming criterion validity. These selected nutrients were also used to develop mathematical cut-point summary scores for each of the three major REAP-S v.2 subscales: Food Sufficiency/Food Insufficiency; Healthy Eating Patterns; and Low Nutrient Density Foods; this allows clinicians to distinguish between healthy and unhealthy dietary patterns with the best sensitivity and specificity. In other words, the cut points allow clinicians to sum scale items within a subscale and use these subscale scores to determine whether a patient generally falls into the healthy or unhealthy dietary range, so the clinician can decide whether to counsel or refer a patient for nutrition counseling. Lower scores reflect poorer dietary patterns. The cut points are *Food Sufficiency/Food Insufficiency*—a score of ≤ 8; *Healthy Eating Patterns* – a score of ≤ 10; *Low Nutrient Density Foods* – a score of ≤ 16; and *Exercise – a score of* ≤ *1.*

### Comparison of REAP-S v.2 to other dietary screeners

We did a PubMed search for validated dietary screeners and found six recent reviews of dietary screeners, with the AHA’s 2020 position paper being the most current and relevant for the US population [1, 18–22].

Reliability and validity were among the theoretical and practice-based factors the AHA 2020 position paper used to evaluate dietary screeners. They included three types of validity: 1) Correspondence between screener scores and a "gold-standard" dietary assessment tool, such as a multiple-day food recall or a semi-quantitative food frequency questionnaire; 2) Generalizability within multiple populations; and 3) Correspondence between screener scores and a measured biomarker. REAP-S v.1 was, over time, validated in all three ways. REAP-S v.2, as a new instrument, has not yet been validated in multiple populations or against a biomarker. Because we retained much of the earlier approach, we suspect it will be generalizable to multiple populations and may well also correlate with biomarkers, such as serum ascorbate levels, as Johnston et al. found the REAP-S v.1 did [23].

However, in addition to validating REAP-S v.2 against the dietary recall, we validated it using several statistically rigorous techniques, such as factor analysis and construct validity. Most other dietary screeners have not undergone this degree of statistical validation. Further, we mathematically established through cluster analysis a marker for participants' objective healthy eating behavior and using ROC curves derived reasonable sensitive and specific cut points for the three main subscales.

The limitations of this study include: 1) The test population was a relatively homogenous, predominantly white population of first-year osteopathic medical students; 2) REAP-S v.2 has not yet been tested on diverse racial, age, educational level and socio-economic groups; 3) As mentioned earlier, REAP-S v.2 has also not yet been tested against a biomarker; 4) Food record data was collected and analyzed by the participants and not via an interview with an experienced research dietitian using the USDA five-step multiple-pass method for dietary recall [24], which may have led to less accurate food records. Another limitation is test–retest reliability assessment as this was not logistically feasible within this study population.

## Conclusion

REAP-S v.2, an updated version of the AHA-recommended original REAP-S, appears to have adequate reliability and has passed multiple types of validity testing. It has been updated to 1) More closely conform to the Dietary Guidelines for Americans 2020–2025; 2) Be easier to administer to patients, including better descriptions of portion sizes for foods; and 3) Provide statistically computed cut points for subscales to assist clinicians in assessing which patients would benefit from nutrition or physical activity counseling.

With respect to clinical utility, REAP-S v.2 is easy to administer and score. It’s also easily converted to an electronic version that can be integrated into an electronic medical record system. It is freely available to clinicians, medical groups, researchers, and medical educators.

## Supplementary Information

**Additional file 1:** **Supplemental Figure 1.** Dendrograms of The Subscale Data Derived from Cluster Analysis. REAP-S v.2 (Rapid Eating Assessment for PARTICIPANTS, Shortened version, v.2) Scale.

## Data Availability

The datasets generated during and/or analyzed during the current study are not publicly available. Questions about access can be directed to the Study P.I.‘s (Drs. Thompson and Segal-Isaacson).

## Abbreviations

AHA: American Heart Association
REAP-S: Rapid Eating Assessment of Participants, Short Version
USDA: United States Department of Agriculture
EFA: Exploratory factor analysis
MAP: Minimum Average Partial Test
ANOVA: Analysis Of Variance
ROC: Receiver Operating Characteristics
PCP: Primary Care Physician
KMO: Kaiser-Meyer-Olkin
SD: Standard Deviation
