# Supplementary material for: Validation and reliability for the updated REAP-S dietary screener, (Rapid Eating Assessment of Participants, Short Version, v.2)
Source: BMC Nutr. 2023 Jul 19;9:88. doi: 10.1186/s40795-023-00747-4 (PMC10357781; doi:10.1186/s40795-023-00747-4)
Supplement: Supplementary file 1 — Additional file 1: Supplemental Figure 1. Dendrograms of The Subscale Data Derived from Cluster Analysis. REAP-S v.2 (Rapid Eating Assessment for PARTICIPANTS, Shortened version, v.2) Scale. [file 40795_2023_747_MOESM1_ESM.docx]

**Supplemental Material** **Supplemental Figure 1: Dendrograms of The Subscale Data Derived from Cluster Analysis**

**b. Healthy Eating Pattern**


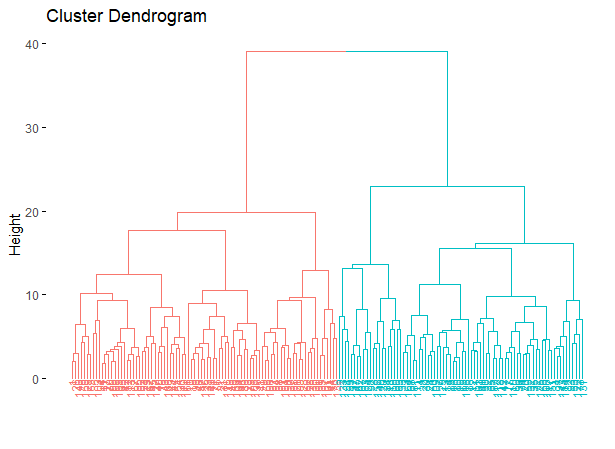


**a. Food Sufficiency/Food Insufficiency**


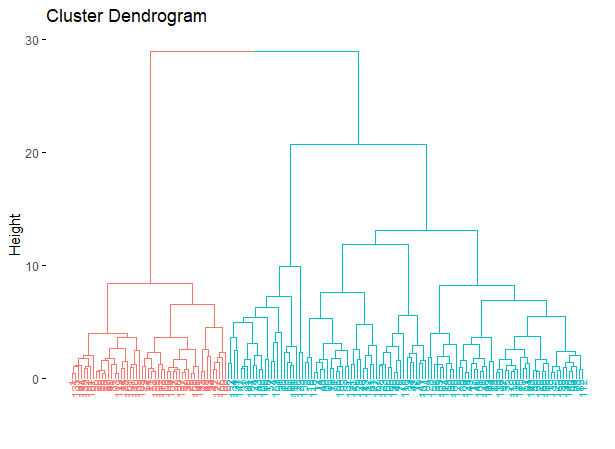


**c. Low Nutrient Density Foods**


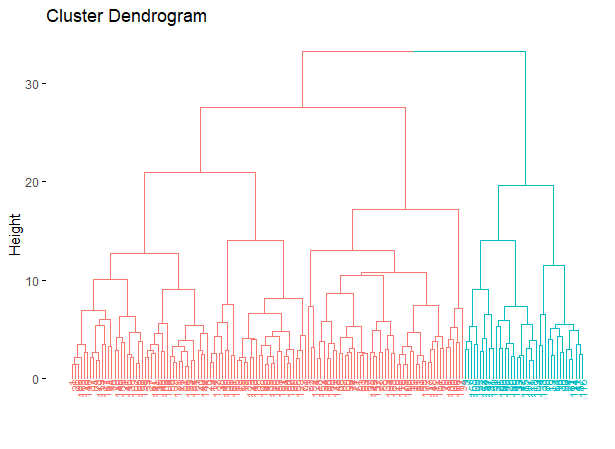


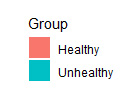


## REAP-S v.2 (Rapid Eating Assessment for PARTICIPANTS, Shortened version, v.2)

**CJ Segal-Isaacson EdD, RD, Kathryn Thompson, PhD, RD, Judy Wylie-Rosett EdD RD. ©2022**

| **In an average week, how often do you:** |  |  |  |  |
| --- | --- | --- | --- | --- |
| 1. Not feel well enough to shop or cook? | **Every day**  (0) | **More than 2 times a week**  (1) | **2 or fewer times a week**  (2) | **Never**  (3) |
| 1. Eat fewer than two meals per day? | **Every day**  (0) | **More than 2 times a week**  (1) | **2 or fewer times a week**  (2) | **Never**  (3) |
| 1. Eat less than 3 ounces per day (see sizes below) of high protein foods such as poultry, meat, fish, tofu, 1 oz. nuts or 1½ cups of beans?   *3 ounces of meat or chicken or fish is the size of a deck of cards or ONE of the following: 1 regular hamburger, 1 chicken thigh or leg, 1 medium filet of fish or a small pork chop, small handful of nuts.* | **Every day**  (0) | **More than 2 times a week**  (1) | **2 or fewer times a week**  (2) | **Never**  (3) |
| 1. Consume less than 2 servings of a calcium-rich food such as milk, yogurt, cheese, calcium-fortified soy, rice or almond milk?   **1 Serving** = 1 cup milk or yogurt; 2 ounces cheese. | **Every day**  (0) | **More than 2 times a week**  (1) | **2 or fewer times a week**  (2) | **Never**  (3) |
| 1. Eat 3 or more servings of vegetables per day?   *(Do not include potatoes and corn as vegetables.)*  **1** **Serving** = ½ cup cooked vegetables or 1 cup leafy raw vegetables.  *1/2 cup is the size of a lightbulb and 1 cup is the size of a baseball.* | **Every day**  (3) | **More than 3 times a week**  (2) | **3 or fewer times a week**  (1) | **Never**  (0) |
| 1. Eat 2 or more servings of fruit per day?   *(Do not include fruit juice or fruit drinks.)*  **1 Serving** = 1/2 cup or 1 medium fruit.  *1/2 cup is the size of a lightbulb.* | **Every day**  (3) | **More than 3 times a week**  (2) | **3 or fewer times a week**  (1) | **Never**  (0) |
| 1. Eat 2 or more servings of whole grain products or high fiber starches a day?   **1 Serving** = 1 slice of 100% whole grain bread or crackers; 1 cup whole grain cereal like Shredded Wheat, Wheaties or oatmeal, ½ cup brown rice or whole wheat pasta, boiled or baked potatoes, yucca, yams or plantain.  *1/2 cup is the size of a lightbulb.* | **Every day**  (3) | **More than 3 times a week**  (2) | **3 or fewer times a week**  (1) | **Never**  (0) |
| 1. Eat fish, shellfish or other seafood? | **Every day**  (3) | **More than 3 times a week**  (2) | **3 or fewer times a week**  (1) | **Never**  (0) |
| 1. Eat beans, peas, lentils or other legumes? | **Every day**  (3) | **More than 3 times a week**  (2) | **3 or fewer times a week**  (1) | **Never**  (0) |
| 1. Eat tree nuts, peanuts or nut butters? | **Every day**  (3) | **More than 3 times a week**  (2) | **3 or fewer times a week**  (1) | **Never**  (0) |
| 1. Use olive oil, peanut oil or other vegetable oils? | **Every day**  (3) | **More than 3 times a week**  (2) | **3 or fewer times a week**  (1) | **Never**  (0) |
| 1. Eat high fat meats such as hamburger, ribs, steak, lamb chops, chicken or turkey wings, hot dogs or cold cuts such as bologna and salami. | **Every day**  (0) | **More than 3 times a week**  (1) | **3 or fewer times a week**  (2) | **Never**  (3) |
| 1. Eat more than 1 tablespoon of cooking or table fats that are solid at room temperature such as butter, stick margarine, bacon fat or vegetable shortening (like Crisco™)?   *1 tablespoon* *is* *the size of a poker chip.* | **Every day**  (0) | **More than 3 times a week**  (1) | **3 or fewer times a week**  (2) | **Never**  (3) |
| 1. Drink 12 ounces or more of non-diet soda, fruit drink/punch, fruit juice or Kool-Aid™ per day?   *1 can of soda = 12 ounces.* | **Every day**  (0) | **More than 3 times a week**  (1) | **3 or fewer times a week**  (2) | **Never**  (3) |
| 1. Eat sweets like cake, cookies, pastries, donuts, toaster pastries, muffins, chocolate and candies | **Every day**  (0) | **More than 3 times a week**  (1) | **3 or fewer times a week**  (2) | **Never**  (3) |
| 1. Eat packaged snack foods such as chips, salted pretzels, pizza bites, etc. | **Every day**  (0) | **More than 3 times a week**  (1) | **3 or fewer times a week**  (2) | **Never**  (3) |
| 1. Eat meals from restaurants, take-out places, convenience stores or entertainment venues? | **Every day**  **(0)** | **More than 3**  **times a week**  **(1)** | **3 or fewer times a week**  (2) | **Never**  **(3)** |
| 1. Prepare meals at home from basic ingredients such as fresh or frozen vegetables, uncooked poultry, pasta, beans etc? | **Every day**  **(3)** | **More than 3 times a week**  (2) | **3 or fewer times a week**  **(1)** | **Never**  **(0)** |
| 1. Have more than 1 alcoholic drink per day if you're a woman or 2 alcoholic drinks per day if you're a man?   **1 Drink** = 5 fluid oz. of wine; 12 fluid oz. of beer, 1½ oz. of hard liquor such as vodka, whiskey, gin, etc. | **Every day**  (0) | **More than 3 times a week**  (1) | **3 or fewer times a week**  (2) | **Never**  (3) |
| 1. Walk for at least one mile (about 2000 steps) or exercise for at least 15 minutes? | **Every day**  (3) | **More than 3 times a week**  (2) | **3 or fewer times a week**  (1) | **Never**  (0) |
| 1. How interested are you in making changes to your eating or physical activity habits in order to be healthier? | Very interested | Interested | Disinterested | Very disinterested |

**SCORING:**

The highest possible score for REAP-S v.2 is 60 points. Although higher numbers generally represent healthier habits, a given person might be high in one subscale and low in another. Therefore we encourage clinicians to focus on subscale scores and perhaps individual scale items rather than a total score for the REAP-S v.2 screener. We also recommend clinicians provide patient education for those specific areas where the person has low or lower scores (and possibly praise the patient for good habits too!).

| Section | Patient’s Score | Action | |
| --- | --- | --- | --- |
|  |  | Investigate/Discuss | Encourage/Commend |
| Food Sufficiency /Food Insufficiency |  | 8 or lower | Greater than 8 |
| Heallthy Eating Pattern |  | 14 or lower | Greater than 14 |
| Low Nutrient Density Foods |  | 16 or lower | Greater than 16 |
| Exercise |  | 1 or lower | Greater than 1 |
| Readiness To Change |  | Disinterested/Very Disinterested | Very Interested/Interested |
